# Experiences of mothers with preterm babies at a Mother and Baby Unit of a tertiary hospital: A descriptive phenomenological study

**DOI:** 10.1002/nop2.373

**Published:** 2019-09-27

**Authors:** Alberta Yemotsoo Lomotey, Victoria Bam, Abigail Kusi‐Amponsah Diji, Ernest Asante, Hannah Boatemaa Asante, Joyce Osei

**Affiliations:** ^1^ Department of Nursing P. M. B., U. P. O. KNUST‐Kumasi Ghana

**Keywords:** experiences, Ghana, mother and baby unit, mothers, preterm babies

## Abstract

**Aim:**

To describe the lived experiences of mothers with preterm babies at a Mother and Baby Unit (MBU) of a tertiary hospital.

**Design:**

A descriptive phenomenological approach.

**Method:**

Ten mothers were purposively sampled during the month of May, 2017 to describe their experiences of having preterm babies. Recorded in‐depth individual interviews were transcribed verbatim; codes were generated and inductively organised into themes.

**Results:**

Four themes were actively generated: ‘Emotional experiences of mothers’, ‘Mother‐baby interaction’, ‘Perception on care and support’ and ‘Challenges within Mother and Baby Unit environment’. Mothers were anxious about the premature delivery and were afraid of possible infant's death. They cherished interactions with their babies during kangaroo mother care and breastfeeding. Mothers applauded the nurses for their professional competence. They expressed concerns about inadequate accommodation, high cost of care, the frequency and duration of mother–baby interactions.

## 1 INTRODUCTION

Preterm babies are those born before the completion of the 37 weeks' gestation, and it is estimated that globally, 15 million babies are born preterm each year (WHO, 2016). About 140,000 (14%) babies are born premature in Ghana annually, and 8,400 of these preterm babies die even before reaching 30 days of their life (UNICEF, 2015). With the rise in the incidence of preterm births year by year, more mothers likewise become affected by the issues associated with preterm delivery (Lee, Long, & Boore, 2009).

## 2 BACKGROUND

The birth of a preterm baby is an unexpected event and often stressful for parents (Aagaard, Uhrenfeldt, Spliid, & Fegran, 2015; Ionio et al., 2016). The baby is usually separated from the mother immediately after birth and is hospitalized in a Neonatal Intensive Care Unit (NICU). The physical separation of the baby, size and appearance of the preterm baby, change in parental role (Al Maghaireh, Abdullah, Chan, Piaw, & Al Kawafha, 2016; Malakouti, Jebraeili, Valizadeh, & Babapour, 2013), condition of infant and tube feeding have been identified as sources of stress for mothers (Valizadeh, Akbarbeglou, & Asad, 2009).

Mothers are usually unprepared psychologically and physically (Coppola & Cassibba, 2010; Payot, Gendron, Lefebvre, & Doucet, 2007) and this may lead to inability to identify with their babies even though they had anticipated becoming mothers (Lasiuk, Comeau, & Newburn‐Cook, 2013; Lindberg & Öhrling, 2008; Ncube, Barlow, & Mayers, 2016). Furthermore, due to the prematurity of babies which makes them less active and almost incapable to give positive responses to maternal alerts, mothers may not have the urge to interact with them (Montirosso, Borgatti, Trojan, Zanini, & Tronick, 2010). Breastfeeding promotes mother–infant interaction (Phuma‐Ngaiyaye & Kalembo, 2016) but could be inhibited in the preterm baby due to inadequate sucking ability, separation between mother and baby, maternal perception of being inadequate and maternal stress due to infant's clinical condition (Giannì et al., 2016). These factors can deprive mothers of the joy of having a baby (Clottey & Dillard, 2013). According to Russell et al. (2014), ensuring participation of mothers in the care and providing emotional support and adequate information are important in the experience of having a preterm baby in the NICU. A meta‐synthesis of 12 qualitative studies of mothers' experiences in the NICU by Aagaard and Hall (2008) thus indicated that being a mother of a preterm baby in a NICU is a process which is attained through close relationship with the baby and healthcare staff.

Several studies (Steyn, Poggenpoel, & Myburgh, 2017; Taylor, 2016; Wigert, Johansson, Berg, & Hellstrom, 2006) described the experiences of mothers with preterm babies in settings where mothers are not accommodated in the hospital premises but can have access to their babies. There is lack of information on the experiences of mothers in a hospital environment that serves as a mother and baby unit (MBU). In this study, the MBU is a unit in the hospital where preterm and other sick babies are admitted. Adjacent to the MBU is an accommodation facility for the mothers to facilitate access to the babies at scheduled intervals. In this situation, mothers may be far away from their homes and require support systems to enable them go through the unexpected event. Understanding the experiences of mothers with preterm babies in this unique environment is a vital step to recognize their needs and address them effectively. Thus, the research question is as follow: What are the experiences of mothers with preterm babies admitted at the mother and baby unit?

## 3 METHODS

### 3.1 Study design

A descriptive phenomenological study was considered appropriate as the researchers were interested in describing the lived experiences of mothers with preterm babies as indicated by Creswell (2007).

### 3.2 Study setting

The study was conducted at the Mother and Baby Unit (MBU) of a tertiary hospital in Ghana. The hospital provides general and specialist services in Medicine, Surgery, Obstetrics and Gynaecology and Child Health. It also serves as a referral centre for both primary and secondary health facilities in Ghana. The MBU has three sections namely: emergency unit, stable baby unit and a preterm unit. Most of the babies admitted at the preterm unit are moderate to late‐term preterm and are not critically ill. The average rate of admission per month at the MBU is about 400, and about 25% of these are preterm babies. The MBU also has an attached room where mothers whose babies are on admission are accommodated and permitted to attend to their babies every 2 hr as part of the hospital's policy. The room has 12 bunk beds which can take 24 mothers but sometimes it is occupied by about 50 women. The average length of stay for moderate to late preterm is 2 weeks.

### 3.3 Study participants

The study population was made up of mothers whose babies were on admission at the preterm unit of the MBU. Mothers with babies born before 37 weeks of gestation were eligible to take part in the study. Mothers whose preterm babies were critically ill and had congenital anomalies were excluded from participation due to the peculiar demands of those conditions on them. Ten mothers were purposively sampled until data saturation was reached. The researchers contacted mothers at the preterm unit and the nature and purpose of the study were explained to them. Appointments were made for individual in‐depth interviews with the mothers who accepted to participate in the research.

### 3.4 Data collection

The in‐depth individual interviews took place in a quiet room adjacent to the preterm unit after written consent had been obtained from participants. A semi‐structured interview guide was used to allow participants to describe their experiences from the birth of the preterm baby through to admission at the preterm unit. During the interview, mothers were asked to narrate what it felt like to have their babies born before term, have the babies admitted at the preterm unit and their stay at the MBU. Prompts were used to elicit more information. The interviews were conducted in Twi (a commonly spoken dialect in Ghana) and recorded using an Easy voice recorder by Digipom. The interviews were conducted by two female midwives who were not working in the unit. They had received training in qualitative research methods. The duration of the interviews ranged from 30–40 min for each participant.

### 3.5 Data analysis

The interviews were transcribed verbatim, translated into English and back translated into Twi by a certified language translator to ensure consistency in the meaning of words. Data collection and analysis occurred concurrently in May 2017 until data saturation was achieved (Morse & Field, 1996). The data were analysed manually using a thematic approach as outlined by Braun and Clarke (2006). The researchers set aside their preconceptions and assumptions about the phenomenon and read through the transcripts several times to get a sense of the data. Codes were generated from the transcribed interviews and grouped into sub‐themes and themes. Direct quotations from participants were used to support the themes.

Trustworthiness was ensured through Lincoln and Guba's (1985) principles of credibility, confirmability, dependability and transferability. Member checking of transcripts and peer checking of themes were undertaken as a method of ensuring credibility and confirmability of the generated themes. Dependability and transferability were achieved through detailed field notes and description of study processes.

### 3.6 Ethical consideration

Ethical approval for the study was obtained from the Committee on Human Research Publication and Ethics (CHRPE) (Ref; CHPRE/AP/329/17) before the commencement of the study. Institutional approval was also obtained from the management of the hospital. Participants were assured of anonymity and confidentiality of information shared. Consent for a 17‐year‐old mother who was included in the study was obtained from an adult family member. Pseudo names were assigned to participants to avoid linkage of the responses to the respondents.

## 4 RESULTS

The respondents' age ranged between 17–38 years. They all had formal education to at least the Junior High School (JHS) level. Nine of the respondents were Christians, and one was a Muslim. Seven of the respondents were married, and three were cohabiting. Seven were employed, and three were unemployed. Six of the respondents had this recent birth as their first delivery. Two of the respondents had twin deliveries while the remaining mothers gave birth to singletons. There were six male babies and six female babies. Five of the mothers gave birth through spontaneous vaginal delivery (SVD) and the other five by caesarean section (CS). The gestational age at which the babies were delivered was between 26–36 weeks. Duration of stay of participants in the MBU at the time of the interviews ranged from 2–21 days. The lived experiences of the participants are described under four themes namely: emotional experiences of mothers, mother–baby interaction, perception on care and support and challenges within MBU environment as seen in Table 1.

**Table 1:** Experiences of mothers with pre‐term babies

Themes	Sub‐themes
Emotional experience of mothers	Fear of the unknown outcome of delivery Sense of guilt Excitement about birth outcomes
Mother–baby interaction	Breastfeeding Skin‐to‐skin contact
Perception on care and support	Availability of competent staff Teaching role of nurses Information sharing on procedures Interpersonal relationship with nurses Mother‐to‐mother support
Challenges within MBU environment	Enforcement of 2‐hr schedule policy High cost of care Inadequate accommodation facilities

Abbreviation: MBU, Mother–Baby Unit.

### 4.1 Theme 1: Emotional experiences of mothers

The mothers expressed different types of emotions depending on the situation they were faced with. They exhibited both negative and positive emotions which were influenced by their thoughts during the period of labour through to the admission of their babies.

#### 4.1.1 Fear of the unknown outcome of delivery

Some of the negative emotions expressed by the mothers were related to fear of unknown outcome of delivery; for instance, losing their babies; and worsening of the condition of their babies. These were expressed as:

I knew I was 8 months pregnant…. I did not understand why I had to deliver at such a period so I got scared…. I was just praying that regardless of his size he would be able to breathe and survive…. [Afua, 25 years]

When I saw my baby in the incubator and the oxygen being administered, I got frightened. I thought my baby's condition had aggravated after delivery but they explained to me that the baby needs that to breathe properly and that dispelled my fears. [Akosua, 18 years]

#### 4.1.2 Sense of guilt

Another negative emotional experience was the feeling of guilt and being responsible for the baby being born prematurely. A mother accentuated:

I felt I caused my baby to be delivered prematurely because I rushed into sexual relationship as a teenager and got pregnant. [Adwoa, 18 years]

#### 4.1.3 Excitement about birth outcomes

Mothers reported some positive experiences that gave them joy and hope. These included baby's sex, observable improvement in baby's condition and twin delivery. They recounted their experiences as:

When l saw my baby after the operation I was very happy. Because I wanted a baby boy and God has given me what I wanted. I was looking for a male child since the first one was a female. [Nana, 36 years]

There are no twins in my family so I was overjoyed to have them (babies). [Awura, 38 years]

I am just happy having this baby regardless of how he is. Babies die daily on this unit so I'm happy whenever I go and meet my baby alive…. some people even have babies with abnormalities yet they are happy. How much more me? Once I see him alive, I become happy and I pray he continues to live. [ Adwoa, 18 years]

Others have delivered preterm babies who have survived so I also have faith that mine too will. [Abena, 26 years]

### 4.2 Theme 2: Mother–baby interaction

Mother and baby interaction started at different times for mothers. There was immediate mother–baby interaction for those who had spontaneous delivery. This interaction process started later for those who had caesarean section but was on the same day of delivery. The first sight of the babies was the starting point where the mothers had to deal with the reality to accept their babies regardless of their characteristics. Some of the mothers had their babies nursed in incubators and thus could not have the privilege to carry the babies in the early days after birth. Activities that emerged as the sub‐themes that described mother–baby interaction were breastfeeding and skin‐to‐skin contact also known as Kangaroo mother care (KMC).

#### 4.2.1 Breastfeeding

Mothers breastfed their babies according to the two‐hourly schedule for seeing their babies. The period of breastfeeding was both rewarding and challenging. Mothers cherished the process of breastfeeding because it promoted mother–baby interaction.

Examples of the rewarding experiences described by mothers were as follows:

I play with my baby and sing for her when breastfeeding. [Akosua 18 years]

I like the feeling when my baby is sucking more than when giving him expressed milk because it makes me feel l have really given birth. [Ama, 27 years]

Other mothers described their experiences of less interaction during breastfeeding:

I do not talk or play with my babies (twins) because they are not grown. [Awura, 38 years]

Although the period of breastfeeding was rewarding, some challenges were expressed by participants. These included babies sleeping for long hours, inability to suck well and pain experienced when expressing breast milk. Examples of these challenges were as follows:

They (twins) sleep a lot so I have to always ensure they wake up to breastfeed else they will just be sleeping. [Yaa, 38 years]

I have joy when breastfeeding although it hurts because my breasts are engorged; I wish he was grown enough to suck better, I would be happier. [Afua, 25 years]

Expressing the breastmilk is painful and my headaches when I am doing it. [Ama, 27 years]

#### 4.2.2 Skin‐to‐skin contact

Mothers who performed KMC enjoyed the close body contact they had with their babies. One mother revealed:

I am happy whenever I place my baby in between my breast. [Akosua, 18 years]

For those who were not performing the KMC, one of them said it was because she had never heard of it whilst the other revealed that she did not do it as she feared hurting the baby in an attempt to position the baby without the support of the nurses. She recounted:

As for KMC I do not do it because I'm afraid the baby would be hurt, when I am positioning him. [Akua, 17 years]

### 4.3 Theme 3: Perception on care and support

The sub‐themes generated from mothers' perceptions on care and support were availability of competent staff, teaching role of nurses, information sharing on procedures, interpersonal relationship with nurses and mother‐to‐mother support.

#### 4.3.1 Availability of competent staff

Mothers were satisfied with the availability of staff and care given to their babies by the health workers.

I'm happy with the care the nurses are providing for my baby. They are always available and administer medications regularly. [Abena, 26 years]

The nurses and doctors take good care of the babies. [Awura, 38 years]

#### 4.3.2 Teaching role of nurses

Some of the mothers mentioned that they had support from the nurses in terms of their teaching role in two main areas namely: how to perform KMC for their babies and position their babies for breastfeeding. Mothers narrated their experiences as follows:

The nurses taught me to do KMC for my babies. They told me because the pregnancy didn't get to term, when I place the babies in between my breast, it's like they are in the warm environment in my womb. [Yaa, 38 years]

I didn't know how to properly position my baby for breastfeeding but the nurses took time to teach me. [Abena, 26 years]

Contrary to the above‐mentioned experiences, a mother did not think the nurses were supportive in meeting her needs. She explained that:

I'm unable to do KMC all alone but when you ask for help from the nurses, they don't provide this. [Akua, 17 years]

#### 4.3.3 Information sharing on procedures

Mothers indicated that though they were often informed of procedures to be carried out on their babies, these were not explained and this sometimes made them anxious especially when they assumed that procedures were being performed on their baby's because their condition was deteriorating. A mother recounted:

They (health workers) usually inform us before performing procedures on our babies but they do not always explain the purpose to us. [Akosua, 18 years]

#### 4.3.4 Interpersonal relationship with nurses

Some mothers described the nurse–mother relationship as cordial whilst others reported it as unfriendly. The quotes below reflect the two different perceptions on the nurse–mother relationship:

The nurses are doing well – they are very approachable. I have not seen any nurse behave in an unfriendly manner towards any mother. [Ama, 27 years expressing a positive view]

No nurse has ever spoken to me. I mean they only speak to you when they are asking you about your baby's folder or when you are going to weigh your baby. [Akua, 17 years expressing a negative view]

It was noted that younger mothers compared with the older mothers complained of unfriendly attitude of some nurses. The oldest among the mothers said that though some nurses were unfriendly, there was no need for the mothers to be offended by the behaviour of the nurses. She explained it as:

We are humans. Someone may meet you the first time and like you while another might not. [Yaa, 38 years]

#### 4.3.5 Mother‐to‐mother support

The hospital provided residential accommodation for the mothers with preterm babies and they lived together in one room. This situation created an opportunity for them to draw support from each other and encouragement as they shared similar experiences. It was found that some of them were able to perform KMC with support from other mothers. One respondent revealed that:

It is not easy to position the baby for KMC but the nurses do not help us so I help one person to position her baby and she also helps with mine. [Akosua, 18 years]

### 4.4 Theme 4: Challenges within MBU environment

The mothers' experiences were not without challenges. They reported some undesirable situations which they had to cope with. These include enforcement of the policy on 2‐hr schedule for seeing their babies, high cost of care and inadequate accommodation facilities.

#### 4.4.1 Enforcement of 2‐hr schedule policy

Most of the respondents wanted more time to interact with their babies and were not happy with the restricted time schedule for seeing their babies every 2 hr. The mothers wished to be with their babies, especially when they were crying.

A mother reported that:

Every day, there are quarrels between some nurses and mothers. This happens when the babies are crying and their mothers want to attend to them but they are prevented by the nurses because the time is not due for them to enter the unit. Sometimes you can just feel your baby is crying because he/she is hungry or has soiled him/ herself but you will not be allowed to see the baby and those times are terrible. [Adwoa, 18 years]

Contrary to this, a mother said she overlooked the restrictions and went to see her baby whenever she felt the need to do so. She stated:

For me, I just walk in whenever I want to see my baby. I don't care about their schedule (meaning the hospital). Who cares about a schedule when my baby is crying? [Ama, 27 years]

#### 4.4.2 High cost of care

Another source of concern to the mothers was the cost of medical care of their babies, which most of them lamented was expensive. All the participants were subscribers to the National Health Insurance Scheme (NHIS), yet they had to buy most of the medications prescribed for their babies because these were not included in the NHIS package. Some concerns about the cost of care were as follows:

I just become scared when I think of how much we would pay by the time we (meaning she and the baby) are discharged. [Adwoa, 18 years]

The NHIS is supposed to be functioning but we buy some of the drugs…., they are quite expensive. [Maame, 20 years]

Some mothers indicated that family members such as their spouses and parents support them financially. A mother reported that:

My husband takes care of the health care finances whilst my mum who is staying with a friend close to the hospital sees to my feeding. [Abena, 26 years]

#### 4.4.3 Inadequate accommodation facilities

Participants were dissatisfied with the accommodation provided for mothers. They described the facility as inadequate because the number of mothers was more than what the room could accommodate. One of the mothers said:

All the mothers cannot fit in the mothers' room here and even the beds are not enough so some of us sleep on the corridor. [Akua, 17 years]

## 5 DISCUSSION

The current study aimed at describing the lived experiences of mothers who had their babies admitted at the preterm unit of the MBU in a tertiary hospital in Ghana.

### 5.1 Emotional experiences of mothers

The unexpected birth of a preterm baby poses immediate as well as long term emotional reactions (Henderson, Carson, & Redshaw, 2016). In this study, mothers expressed mixed reactions of anxiety, joy and guilt as corroborated by Arnold et al. (2013). Anxiety was as a result of the perceived deterioration of the condition of the baby and fear of the infant dying, consistent with the findings of Lasiuk et al. (2013). Mothers felt guilty for giving birth to a preterm baby similar to the findings of earlier studies (Bernard et al., 2011; Lasiuk et al., 2013; Taylor, 2016). These emotional reactions experienced by mothers can have adverse effect on maternal health and mother–infant relationship (Milgrom et al., 2008); thus, there is a need for healthcare staff to provide emotional support for such mothers.

Although the mothers in the current study were unprepared for the birth of a preterm baby, some were happy as result of the survival of the baby, sex of the baby and absence of congenital anomalies and this agrees with Arnold et al. (2013). The excitement associated with the sex of the baby was particularly linked to male babies. This might be related to the belief that women are likely to gain recognition by their husbands and families when they give birth to a son (Shah, 2005). Hesketh and Zhu (2006) posited that sons continue the family line, earn more income and take responsibility in caring for parents in sickness and in old age similar to what is seen in patriarchal traditions in most parts of Ghana. According to Tanner, Sabrine, and Wren (2005), preterm babies with congenital anomalies may require surgical intervention and long period of hospitalization which is likely to place considerable burden and stress on mothers. Mothers in this study were happy to have preterm babies with no congenital anomalies. Health staff when providing emotional support could focus on some of these factors which made mothers happy to enable them cope with the stress of caring for a preterm infant.

### 5.2 Mother–baby interaction

In this study, breastfeeding and KMC provided opportunity for mothers to exhibit maternal responsive behaviours such as cuddling their babies, talking to them, playing with them, smiling at them and watching for the response of their babies to these actions, consistent with the findings of Amankwaa, Pickler, and Boonmee (2007). Inadequate sucking ability and infant drowsiness reported as challenges encountered in breastfeeding did not prevent mothers from breastfeeding their babies contrary to the findings of Giannì et al. (2016). This was because, breastfeeding was seen as a time to have contact with the baby, a positive bonding experience and the joy of motherhood as corroborated by Kair, Flaherman, Newby, and Colaizy (2015) and Flacking, Ewald, Nyqvist, and Starrin (2006).

Some mothers did not have the urge to actively interact with their babies during breastfeeding because they were unable to respond to their maternal alerts as suggested by Montirosso et al. (2010). It has also been reported that mothers with high‐stress levels have less interaction with their babies (Clottey & Dillard, 2013) and may not practice skin‐to‐skin contact (Gonya & Nelin, 2013). Staff caring for preterm babies should identify the specific needs of mothers, educate them on stress relief and promote breastfeeding and KMC activities to enhance mother–baby interaction for healthy growth and development of infants (McEwen, 2006).

### 5.3 Perception on care and support

Nurses play a major role in providing education and support for mothers based on their unique needs (Cervantes, Freeley, & Lariviere, 2011). In the current study, mothers expressed confidence in the healthcare personnel and indicated that they received support from the nursing staff in feeding and handling their babies. However, a mother also indicated that she had to seek assistance from another mother to practice KMC. The need to encourage peer support to increase the confidence of mothers and complement the efforts of nursing staff is vital (Cooper et al., 2007; Hurst, 2006), but this should be supervised by health staff when the support is offered in the premises of the hospital.

Some mothers described their interactions with the nurses as cordial whilst others described it as unfriendly. They perceived that some of the nurses were unfriendly because they felt these nurses did not show concern for their well‐being. It has been reported that the traumatic experience of having a preterm birth is anticipated to upsurge the need for emotional support and encouragement from healthcare staff (Sawyer et al., 2013). According to De Rouck and Leys (2009) though preterm infants receive adequate care, mothers are often left to handle their own emotions due to the workload of NICU staff and this was not purposeful. Nurses working in such units need to acknowledge the challenges experienced by mothers and plan effective care to meet the needs of both baby and mother. Provision of timely and comprehensive information to mothers on the condition, treatment and care of their babies (Gaucher & Payot, 2011; Steyn et al., 2017) could relieve mothers' anxiety and also promote confidence in the healthcare team (Arzani, Valizadeh, Zamanzadeh, & Mohammadi, 2015; Williams et al., 2018).

### 5.4 Challenges within MBU environment

Mothers disliked the MBU policy of seeing their babies only at fixed time schedules. This regulation inhibited them from having access to their babies whenever they wanted and this is consistent with other studies (Baird, Davies, Hinds, Baggott, & Rehm, 2015; Williams et al., 2018). According to Taylor (2016), feeling of separation and lack of control could be detrimental to the well‐being of mothers. Allowing mothers to stay with their babies most of the day except when medical rounds and procedures are being carried out could be explored to boost their morale and sense of control.

Mothers in this study were worried about the high cost of care which corroborates the findings of Hodek Schulenburg and Mittendorf (2011) that the birth of a preterm baby has significant financial implications on parents. The extended length of stay in hospital and prescription of drugs that are not covered under health insurance are major cost factors. Measures to reduce the cost of care could offer relief to families.

The birth and care of a preterm baby are stressful and maternal stress contributes to poor sleep quality and depression which can result in poor health‐related quality of life (Lee & Hsu, 2012). The provision of accommodation for mothers in the hospital is ideal, but there is a need to ensure that the facility is spacious and comfortable to promote adequate rest and sleep.

## 6 LIMITATIONS

Some aspects of the experiences of participants on the birth and care of the preterm baby could have been missed based on the depth of information participants were willing to share. However, the results provide guidance for nurses managing preterm babies.

## 7 IMPLICATIONS FOR PRACTICE

Nurses working in mother and baby units need to acknowledge the challenges faced by mothers, particularly, inadequate information on the care of the preterm baby and emotional demands. This will enable them to plan effective care to meet the needs of both baby and mother. There is a need for health staff to identify the determinants of positive experience and also use strategies such as mother‐to‐mother support to enable mothers cope with the care of the preterm baby.

## 8 CONCLUSION

The birth of a preterm baby is unexpected and associated with feelings of anxiety, fear and inability to have frequent access and interact with the infant during the period of hospitalization in the preterm unit. Involvement of mothers through information sharing, education on rationale for care and hospital policies is essential. Cordial nurse–mother interaction and provision of support in areas such as breastfeeding and KMC would empower mothers and promote their physical and psychological well‐being. Expanding the benefit package of the national health insurance scheme to cover specific medical expenses of preterm infants might address the financial concerns of mothers. A decent and spacious accommodation at the MBU is important to ensure that mothers have adequate rest and comfort to enable them cope with the unplanned event of preterm birth. Research to consider the needs of MBU staff in rendering care to preterm babies and support to mothers could generate evidence that will ensure holistic service delivery in mother and baby units.

## CONFLICT OF INTEREST

The authors declare that there is no conflict of interest.

## AUTHOR'S CONTRIBUTIONS

AYL: designed the study, collected data, performed data analysis and wrote the first draft of the paper. VB and AK‐AD: contributed to the study design assisted with data analysis, critically reviewed and revised the final draft. EA: contributed to the study design, critically reviewed and revised the final draft of the paper. HBA and JO: contributed to the study design, collected data, critically reviewed and revised the final draft of the paper. The manuscript has been read and approved for publication by all authors.
